# Progression Patterns and Post-Progression Survival in Recurred Intrahepatic Cholangiocarcinoma Patients: A Novel Prognostic Nomogram Based on Multicenter Cohorts

**DOI:** 10.3389/fonc.2022.832038

**Published:** 2022-04-08

**Authors:** Chongyu Zhao, Chaobin He, Jiawei Lu, Xin Huang, Cheng Chen, Xiaojun Lin

**Affiliations:** ^1^ Department of Pancreatobiliary Surgery, State Key Laboratory of Oncology in South China, Collaborative Innovation Center for Cancer Medicine, Sun Yat-sen University Cancer Center, Guangzhou, China; ^2^ Department of Oncology, The Second Hospital of Dalian Medical University, Dalian, China; ^3^ Department of Cardiology, The First Affiliated Hospital of Dalian Medical University, Dalian, China

**Keywords:** intrahepatic cholangiocarcinoma, post-progression survival, recurrence, prognosis, nomogram

## Abstract

**Background:**

The post-progression survival (PPS) of recurred intrahepatic cholangiocarcinoma (iCCA) patients relates to the characteristics of tumor progression. Moreover, the prediction model of PPS in those patients has not been well established. This study aimed at developing a novel nomogram for predicting PPS in recurred iCCA patients.

**Method:**

Clinical characteristics were retrospectively collected in 396 patients diagnosed with iCCA from cohorts of Sun Yat-sen University Cancer Center (SYSUCC) and the First Hospital of Dalian Medical University (FHDMU). The PPS in patients with different progression patterns was investigated. The nomogram of PPS was established with the Cox regression model in the primary cohort. Then the nomogram was verified in the external validation cohort.

**Results:**

Liver progression was the commonest pattern (42.08%) in recurred iCCA patients, while patients with local LN progression had significantly better PPS than those with other patterns. The independent prognostic factors comprised elevated CEA levels, tumor differentiation, N stage 8th, adjuvant therapy, Local LN metastasis, Liver Metastasis only, and Multiple Metastasis. The nomogram constructed on these factors achieved satisfied C-indexes of 0.794 (95% CI 0.769–0.828) and 0.827 (0.779–0.876) for the training and validation cohorts, respectively. These values were significantly higher than those of the 8th TNM stage system (all p < 0.001). The recurred iCCA patients could be precisely classified into high- and low-risk groups according to the cutoff point of this nomogram (p < 0.01).

**Conclusion:**

The investigation of progression patterns and the development of this nomogram can offer new evidence to precisely postoperative and post-progression management of iCCA patients.

## Introduction

Intrahepatic cholangiocarcinoma (iCCA) is composed of heterogeneous malignancies that originate from, hence displaying pathological characteristics of the biliary track, or trans-differentiate from hepatocytes (1, 2). Radical surgical resection offers the only treatment option that increases potential long-term survival for iCCA patients. It has been reported in numerous studies that overall survival of iCCA patients ranges from 17% to 42% after surgery (3–6), resulting in the rise of recurrence rates in these patients. In patients with 26-month median disease-free survival, recurrence rates were reported to be up to 50%–60% (7).

Additionally, the outcomes of recurred iCCA patients could be determined by various factors. In a previous study in patients with recurrence in pancreatic ductal adenocarcinoma, it was observed that the patients with multiple metastases showed significantly distinct outcomes as compared to those with “liver only” metastasis, suggesting that different survival outcomes in patients could be resulted from different progression patterns (8). Moreover, in the efforts of developing effective adjuvant therapy for iCCA patients, treatments after operation were proven to improve the outcomes in patients with recurrence (9). Consequently, the number of patients with post-progression survival (PPS) have been accounting for an increasing proportion in overall survival (OS), and the vital role of post-progression survival in recurred iCCA patients is now well-established. Even though there were several predictive stage systems estimating the OS or progression-free survival (PFS) of iCCA patients, to date there remains no established model to predict the outcome for patients in the category of PPS (10). To better stratify the patients for precise medical intervention, it would be instrumental to construct a predictive system of PPS in iCCA patients.

Herein, in the present study, we sought to compare the PPS in iCCA patients with different progression patterns in multicenter patient cohorts and establish a prognostic nomogram to predict the PPS of iCCA patients after radical surgical resection based on multicenter cohorts.

## Method

### Patients’ Characteristics

396 consecutive patients pathologically diagnosed with iCCA who underwent radical surgical resection at Sun Yat-sen University Cancer Center (SYSUCC) and the First Affiliated Hospital of Dalian Medical University (FHDMU) were preliminarily enrolled in the present study (289 patients from SYSUCC between January 2000 and December 2018 as the primary cohort and 107 patients from FHDMU between May 2013 and December 2019 as the validation cohort). Variables of the total patients related to preoperative baseline characteristics, liver function, tumor marker, pathological diagnosis, tumor progression, and time to death or last visit were collected from the medical record, as shown in **Supplementary Table 1**. The two cohorts of this study owned the same indications and contraindications to resection. Then a total of 280 recurred patients were finally enrolled in the recurrence cohort (200 patients from the primary cohort and 78 patients from the validation cohort). Previously researched inflammation-based indexes were calculated and analyzed as well. This study obtained the written informed consent from all the patients and was approved by the ethics committees of two participating centers.

### Follow-up and Survival Outcomes

The routine postoperative follow-up began at 30 days after resection, then each 3 months for the first year and 6 months until death or dropout. Patterns and timing of recurrence were obtained at regular follow-up, which consisted of regular abdominal CT, carcinoembryonic antigen (CEA) measurement, and carbohydrate antigen 19-9 (CA19-9) measurement. “Elevated CEA” was defined as CEA value >5ng/ml; other threshold values of those characteristics are exhibited in **Supplementary Table 1**. Additional imaging examinations were conducted to determine patterns of recurrence in necessary. Follow-up data of two cohorts were retrieved on November 30, 2020. The outcome variables of this study, PPS, were calculated from the date of tumor progression to the date of death or last follow-up.

### Progression Patterns

Imaging findings were the primary methods to confirm the progression patterns. While the imaging findings were ambiguous about recurrence or progression, biopsy was conducted. The progression patterns were described by the first location of recurrence. The demarcation point distinguishing early and late progression was defined as 2 years after surgical resection as previous studies (11). The term “Local LN” referred to local lymph-node metastasis. The term “Liver” referred to isolated hepatic recurrence, while the term “Multiple” referred to multiple metastases.

### Statistical Analysis

Categorical variables were analyzed in whole numbers and proportions. Proportions were compared using the chi-square test or the Fisher exact test. The Mann–Whitney U test was conducted to compare the distributions of continuous variables. Survival curves were calculated using the Kaplan–Meier method and then compared with the log-rank test. The multivariable analysis of the predictive factors of PPS was performed using the Cox regression model. Then the nomogram was constituted based on the multivariable analysis in the training cohort. The predictive performance was measured by Harrell’s concordance index (C-index) and assessed with calibration curves and survival curves. SPSS software version 22 (SPSS Inc., Chicago, IL, USA) and R software version 4.1.1 (R Development Core Team; http://www.r-project.org) were used. All statistical inferences were based on two-sided p values, with values <0.05 taken to indicate statistical significance. Particularly, those variables which had p < 0.2 in the univariable analysis were included in the multivariable regression analysis.

## Results

### Characteristics of Patients

The preoperative clinical, surgical, and postoperative pathological demographics of the recurred iCCA patients in the primary and validation cohorts are exhibited in **Table 1**. The primary cohort consisted of 111 (38.4%) female and 178 (61.6%) male patients with a median age of 56 years. A total of 130 (45.0%) patients had received chemotherapy after resection. As for the validation cohort, 45 females (42.1%) and 62 males (57.9%) with a median age of 64 years were enrolled. Thirty-five (32.7%) patients had received chemotherapy after resection. There were no significant differences in baseline characteristics between the two cohorts.

**Table 1:** Clinical and pathological characteristics of recurred iCCA patients in the primary cohort (SYSUCC cohort) and validation cohort (FHDMU cohort).

Variables	Primary cohort (n = 202)	Validation cohort (n = 78)	Variables	Primary cohort (n = 202)	Validation cohort (n = 78)
Gender			mGPS		
Male	68 (33.7%)	33 (42.3%)	0	152 (75.2%)	26 (33.3%)
Female	134 (66.3%)	45 (57.7%)	1	47 (23.3%)	31 (39.7%)
Age (years)			2	3 (1.49%)	21 (26.9%)
≤60 years	74 (36.6%)	53 (67.9%)	Microvascular invasion		
>60 years	128 (63.4%)	25 (32.1%)	Absence	159 (78.7%)	69 (88.5%)
Progression period			Presence	43 (21.3%)	9 (11.5%)
Early	149 (73.8%)	56 (71.8%)	Lymph-vessel invasion		
Late	16 (7.92%)	8 (10.3%)	Absence	187 (92.6%)	–
Medium	37 (18.3%)	14 (17.9%)	Presence	15 (7.43%)	–
Progression patterns			Macrovascular invasion		
Liver	85 (42.1%)	29 (37.2%)	Absence	187 (92.6%)	69 (88.5%)
Liver+Local	19 (9.41%)	11 (14.1%)	Presence	15 (7.43%)	9 (11.5%)
Local	41 (20.3%)	8 (10.3%)	Satellite sites		
Multiple	40 (19.8%)	23 (29.5%)	Absence	125 (61.9%)	77 (98.7%)
Others	17 (8.42%)	7 (8.97%)	Presence	77 (38.1%)	1 (1.3%)
WBC count (×10^9^/L)			Adjacent organ invasion		
≤10	176 (87.1%)	66 (84.6%)	Absence	173 (85.6%)	75 (96.2%)
>10	26 (12.9%)	12 (15.4%)	Presence	29 (14.4%)	3 (3.85%)
HGB (g/L)			Tumor size		
≤175	17 (8.42%)	22 (28.2%)	≤5cm	64 (31.7%)	35 (44.9%)
>175	185 (91.6%)	56 (71.8%)	≤5cm	138 (68.3%)	43 (55.1%)
PLT (×109/L)			LN metastasis		
≤350	195 (96.5%)	73 (93.6%)	Absence	162 (80.2%)	70 (89.7%)
>350	7 (3.47%)	5 (6.41%)	Presence	40 (19.8%)	8 (10.3%)
ALT (U/L)			Positive LN number:		
≤50	159 (78.7%)	41 (52.6%)	0	162 (80.2%)	70 (89.7%)
>50	43 (21.3%)	37 (47.4%)	1	17 (8.42%)	2 (2.56%)
AST (U/L)			2	10 (4.95%)	1 (1.28%)
≤40	171 (84.7%)	43 (55.1%)	4	6 (2.97%)	2 (2.56%)
>40	31 (15.3%)	35 (44.9%)	5	3 (1.49%)	2 (2.56%)
ALP (U/L)			6	3 (1.49%)	–
≤125	110 (54.5%)	16 (20.5%)	>6	1 (0.50%)	1 (1.28%)
>125	92 (45.5%)	62 (79.5%)	Tumor differentiation		
GGT (U/L)			Low	24 (11.9%)	2 (2.56%)
≤60	63 (31.2%)	10 (12.8%)	Medium/high	178 (88.1%)	76 (97.4%)
>60	139 (68.8%)	68 (87.2%)	T stage 8th		
ALB (g/L)			1	18 (8.91%)	62 (79.5%)
>40	4 (1.98%)	27 (34.6%)	2	22 (10.9%)	3 (3.85%)
≤40	198 (98.0%)	51 (65.4%)	3	141 (69.8%)	10 (12.8%)
TBIL (μmol/L)			4	21 (10.4%)	3 (3.85%)
≤20.5	180 (89.1%)	42 (53.8%)	N stage 8th		
>20.5	22 (10.9%)	36 (46.2%)	Absence	162 (80.2%)	70 (89.7%)
IBIL (μmol/L)			Presence	40 (19.8%)	8 (10.3%)
≤15	187 (92.6%)	51 (65.4%)	TNM 8th		
>15	15 (7.43%)	27 (34.6%)	IA	18 (8.91%)	24 (30.8%)
HBsAg			IB	21 (10.4%)	35 (44.9%)
Absence	108 (53.5%)	–	II	24 (11.9%)	2 (2.56%)
Presence	94 (46.5%)	–	IIIA	84 (41.6%)	6 (7.69%)
CA19-9 (U/mL)			IIIB	55 (27.9%)	11 (14.1%)
≤35	81 (40.1%)	20 (25.6%)	After operation therapy		
>35	121 (59.9%)	58 (74.4%)	Absence	78 (38.6%)	51 (65.4%)
CEA (ng/mL)			Presence	124 (61.4%)	27 (34.6%)
≤5	139 (68.8%)	43 (55.1%)	LN7 metastasis		
>5	63 (31.2%)	35 (44.9%)	Absence	198 (98.0%)	78 (100%)
NLR			Presence	4 (1.98%)	0 (0%)
<2.62	124 (61.4%)	24 (30.8%)	LN8 metastasis		
≥2.62	78 (38.6%)	54 (69.2%)	Absence	194 (96.0%)	73 (93.6%)
PLR			Presence	8 (3.96%)	5 (6.41%)
<104.85	117 (57.9%)	17 (21.8%)	LN9 metastasis		
≥104.85	85 (42.1%)	61 (78.2%)	Absence	196 (97.0%)	–
SII			Presence	6 (2.97%)	–
0	47 (23.3%)	17 (21.8%)	LN12 metastasis		
1	155 (76.7%)	61 (78.2%)	Absence	177 (87.6%)	68 (87.2%)
LCR			Presence	25 (12.4%)	10 (12.8%)
0	12 (5.94%)	–	LN13 metastasis		
1	190 (94.1%)	–	Absence	194 (96.0%)	74 (94.9%)
PNI			Presence	8 (3.96%)	4 (5.13%)
0	189 (93.6%)	36 (46.2%)	LN14 metastasis		
1	13 (6.44%)	42 (53.8%)	Absence	201 (99.5%)	–
PI			Presence	1 (0.50%)	–
0	143 (70.8%)	24 (30.8%)	LN16 metastasis		
1	50 (24.8%)	44 (56.4%)	Absence	199 (98.5%)	–
2	9 (4.46%)	10 (12.8%)	Presence	3 (1.49%)	–

The general survival outcomes were as follows: in the primary cohort, the 1-, 2-, and 3-year OS rates were 78.2%, 64.9%, and 52.2%, respectively; the 1-, 2-, and 3-year PFS were 48.5%, 35.4%, and 31.5% while the 1-, 2-, 3-year PPS were 49.6%, 30.5%, and 19.8%, respectively; in the validation cohort, the 1-, 2-, 3-year OS rates were 61.8%, 40.4%, and 32.7% and the 1-, 2-, and 3-year PFS were 44.7%, 29.3%, and 21.0% while the 1-, 2-, and 3-year PPS were 53.8%, 24.3%, and 2.6%, respectively.

### Comparisons of PPS Classified by Progression Patterns

**Figure 1:**
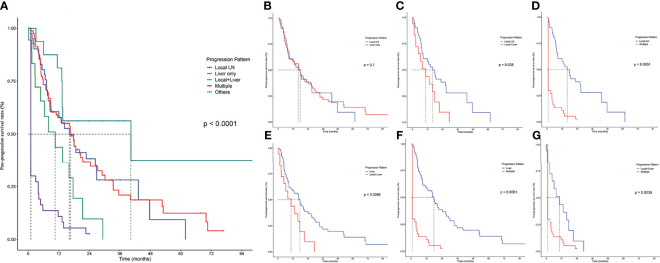
Pairwise comparison of post-progression survival in patients with different progression patterns. **(A)** PPS stratified by all progression patterns. Stratification of patients by comparing the following progression patterns: **(B)** Local LN vs. Liver only; **(C)** local LN vs. Local+Liver; **(D)** local LN vs. Multiple; **(E)** Liver vs. Local+Liver; **(F)** Liver vs. Multiple; **(G)**, Local+Liver vs. Multiple.

The progression patterns of iCCA patients after surgery were classified into 5 subgroups (**Figure 1A**): Local LN, Liver only, Local plus Liver, Multiple, and Others. Particularly, there were 17 patients in the “Others” group, including 10 patients with isolated lung metastasis, 4 patients with single distant metastasis, and 2 patients with other non-typical metastasis. Although the most optimistic survival curve was observed in this group, the “Others” progression pattern was not included in the subsequent analysis because of its heterogeneity and minor sample size.

Liver progression was the commonest pattern since there were 85 (42.08%) patients who had isolated hepatic progression, followed by Local progression (41 patients, 20.30%) and Multiple progression (40 patients, 19.80%), while Local plus Liver was the rarest pattern (19 patients, 9.41%). Patients with liver progression had the longest median PPS of 17.70 months (95% CI 12.476–22.924), followed by patients with Local LN metastasis (median PPS 16.13 months, 95% CI 9.021–23.246) and Local plus Liver progression (median PPS 10.57 months, 95% CI 3.266–17.867).

The comparisons of PPS between Local LN and Liver showed no significant differences (**Figure 1B**); apart from this result, Local LN had significantly higher survival rates (p < 0.05) than Local plus Liver (**Figure 1C**) and Multiple (**Figure 1D**). In addition, the comparisons of PPS between Liver and Local plus Liver (**Figure 1E**), between Liver and Multiple (**Figure 1F**), and between Local plus Liver and Multiple (**Figure 1G**) revealed that the former owned a higher PPS rate (p < 0.05) than the latter. Overall, patients with local LN progression had significantly higher survival rates than those with other progression patterns. On the contrary, multiple metastases corresponded with the poorest rates among these patterns.

### Prognostic Factors of PPS

**Figure 2:**
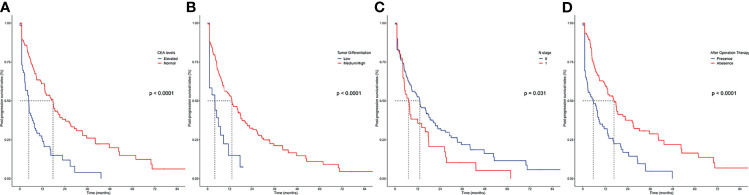
Kaplan–Meier curves for post-progression survival in patients with iCCA in the SYSUCC cohort stratified by the significant prognostic factor defined by the Cox-regression analysis. **(A)** CEA levels; **(B)** tumor differentiation; **(C)** N stage; **(D)** after operation therapy.

The primary cohort was employed to conduct univariable and multivariable analyses and further establish the predictive nomogram. The univariable analysis identified 16 factors significantly correlating to PPS (**Table 2**). The Cox-regression analysis was conducted on the basis of univariable analysis. As a result, the multivariable analysis defined CEA (HR, 2.102; 95% CI 1.318–3.071; p < 0.001), tumor differentiation (HR, 6.125; 95% CI 1.228–30.456; p = 0.027), N stage 8th (HR, 6.077; 95% CI 0.680–54.274; p = 0.036), after-operation therapy (HR, 0.474; 95% CI 0.315–0.713; p < 0.001), Local LN metastasis (HR, 0.938; 95% CI 0.852–1.291; p = 0.041), Liver Metastasis only (HR, 0.881; 95% CI 0.709–1.093; p = 0.039), and Multiple Metastases (HR, 0.434; 95% CI 0.335–0.561; p < 0.001) as independent prognostic factors of PPS (**Table 2**). The PPS survival curves stratified by these factors are shown in **Figure 2**.

**Table 2:** Univariate and multivariate analysis of prognostic factors of PPS.

Characteristics	Levels	PPS						
		Univariate analysis			Multivariate analysis			
		HR	95%	p	HR	95%		p
Age (years)	≤60 years	Reference						
	>60 years	1.090	0.784–1.516	0.609				
Gender	Male	Reference						
	Female	1.041	0.744–1.456	0.814				
WBC count (×10^9^/L)	≤10	Reference			Reference			
	>10	2.006	1.296–3.105	0.002	0.872	0.384–1.976	0.742	
HGB (g/L)	≤175	Reference						
	>175	1.228	0.662–2.277	0.515				
PLT (×109/L)	≤350	Reference						
	>350	0.542	0.239–1.232	0.144	1.632	0.513–5.194	0.407	
ALT (U/L)	≤50	Reference						
	>50	1.242	0.846–1.824	0.269				
AST (U/L)	≤40	Reference						
	>40	0.976	0.620–1.546	0.917				
GGT (U/L)	≤60	Reference			Reference			
	>60	1.599	1.111–2.303	0.012	1.120	0.177–5.688	0.989	
ALP (U/L)	≤125	Reference			Reference			
	>125	2.035	1.463–2.833	<0.001	1.316	0.851–2.036	0.217	
ALB (g/L)	>40	Reference			Reference			
	≤40	1.200	0.978–1.472	0.081	1.135	0.797–1.614	0.483	
TBIL (mmol/L)	≤20.5	Reference						
	>20.5	1.292	0.778–2.147	0.323				
IBIL (mmol/L)	≤15	Reference						
	>15	0.901	0.473–1.714	0.750				
NLR	<2.62	Reference			Reference			
	≥2.62	1.432	1.030–1.990	0.033	0.776	0.473–1.273	0.315	
LMR	<4.06	Reference			Reference			
	≥4.06	0.786	0.569–1.087	0.145	0.902	0.579–1.405	0.648	
PLR	<104.85	Reference			Reference			
	≥104.85	1.371	0.988–1.903	0.059	1.167	0.753–1.809	0.489	
SII	0	Reference			Reference			
	1	1.435	0.975–2.112	0.067	1.121	0.662–1.899	0.670	
LCR	0	Reference						
	1	0.943	0.495–1.796	0.858				
PNI	0	Reference						
	1	0.880	0.448–1.730	0.712				
PI				<0.001			0.534	
	0	Reference			Reference			
	1	0.500	0.241–1.036	0.062	0.587	0.112–3.085	0.529	
	2	1.146	0.537–2.444	0.724	0.992	0.326–3.016	0.989	
mGPS				<0.001			0.996	
	0	Reference			Reference			
	1	0.811	0.199–3.299	0.770	1.053	0.148–7.512	0.959	
	2	1.709	0.412–7.083	0.460	1.012	0.177–5.788	0.989	
HBsAg	Absence	Reference			Reference			
	Presence	0.762	0.550–1.055	0.101	0.716	0.459–1.118	0.142	
CA 19-9 (U/mL)	≤35	Reference						
	>35	1.190	0.856–1.656	0.301				
CEA (ng/mL)	≤5	Reference			Reference			
	>5	2.640	1.866–3.735	<0.001	1.807	1.147–2.847	0.011	
Microvascular invasion	Absence	Reference						
	Presence	1.049	0.704–1.563	0.815				
Lymph-vessel invasion	Absence	Reference						
	Presence	1.201	0.691–2.088	0.516				
Macrovascular invasion	Absence	Reference						
	Presence	1.417	0.764–2.628	0.268				
Satellite sites	Absence	Reference						
	Presence	1.216	0.876–1.690	0.243				
Adjacent organ invasion	Absence	Reference						
	Presence	1.106	0.695–1.760	0.670				
Tumor size	≤5 cm	Reference			Reference			
	≤5 cm	2.376	1.615–3.495	<0.001	1.424	0.856–2.366	0.173	
Liver capsule invasion	Absence	Reference						
	Presence	0.880	0.631–1.228	0.452				
Tumor differentiation	Low	Reference			Reference			
	Medium/High	5.926	1.425–24.641	0.014	1.059	0.694–1.615	0.038	
T stage 8th				0.104			0.822	
	1	Reference			Reference			
	2	1.149	0.582–2.269	0.689	1.674	0.148–18.987	0.677	
	3	1.316	0.701–2.470	0.393	1.381	0.354–5.387	0.642	
	4	0.953	0.548–1.658	0.864	1.308	0.383–4.462	0.668	
N stage 8th	Absence	Reference			Reference			
	Presence	1.537	1.049–2.250	0.027	5.793	0.364–8.118	0.041	
TNM 8th				0.041			0.074	
	I	Reference			Reference			
	II	0.948	0.547–1.645	0.850	1.452	0.109–19.306	0.778	
	IIIa	0.999	0.589–1.693	0.996	2.335	0.462–11.799	0.305	
	IIIb	0.687	0.464–1.017	0.060	1.381	0.316–6.031	0.667	
After operation therapy	Absence	Reference			Reference			
	Presence	0.496	0.357–0.690	<0.001	0.521	0.343–0.791	0.002	
Local LN metastasis	Absence	Reference			Reference			
	Presence	1.116	0.913–1.365	0.013	0.710	0.313–1.613	0.037	
Liver metastasis only	Absence	Reference			Reference			
	Presence	1.246	1.056–1.471	0.009	1.236	0.627–2.439	0.041	
Local+Liver Metastasis	Absence	Reference			Reference			
	Presence	0.811	0.614–1.070	0.139	1.559	0.583–4.167	0.376	
Multiple metastasis	Absence	Reference			Reference			
	Presence	0.480	0.395–0.582	<0.001	5.911	2.742–12.744	<0.001	
Progression period	Early	Reference						
	Late	1.240	0.850–1.810	0.264				
LN7 metastasis	Absence	Reference						
	Presence	1.114	0.274–4.533	0.880				
LN8 metastasis	Absence	Reference			Reference			
	Presence	1.447	0.834–2.509	0.188	1.026	0.387–2.716	0.960	
LN9 metastasis	Absence	Reference			Reference			
	Presence	1.998	0.814–4.906	0.131	1.181	0.280–4.978	0.821	
LN12 metastasis	Absence	Reference			Reference			
	Presence	1.462	1.065–2.008	0.019	1.281	0.721–2.276	0.399	
LN13 metastasis	Absence	Reference						
	Presence	0.953	0.420–2.162	0.907				
LN14 metastasis	Absence	Reference						
	Presence	0.049	0.000–526.981	0.525				
LN16 metastasis	Absence	Reference						
	Presence	1.238	0.394–3.893	0.714				

### Construction and Validation of Nomogram for PPS Prediction

**Figure 3:**
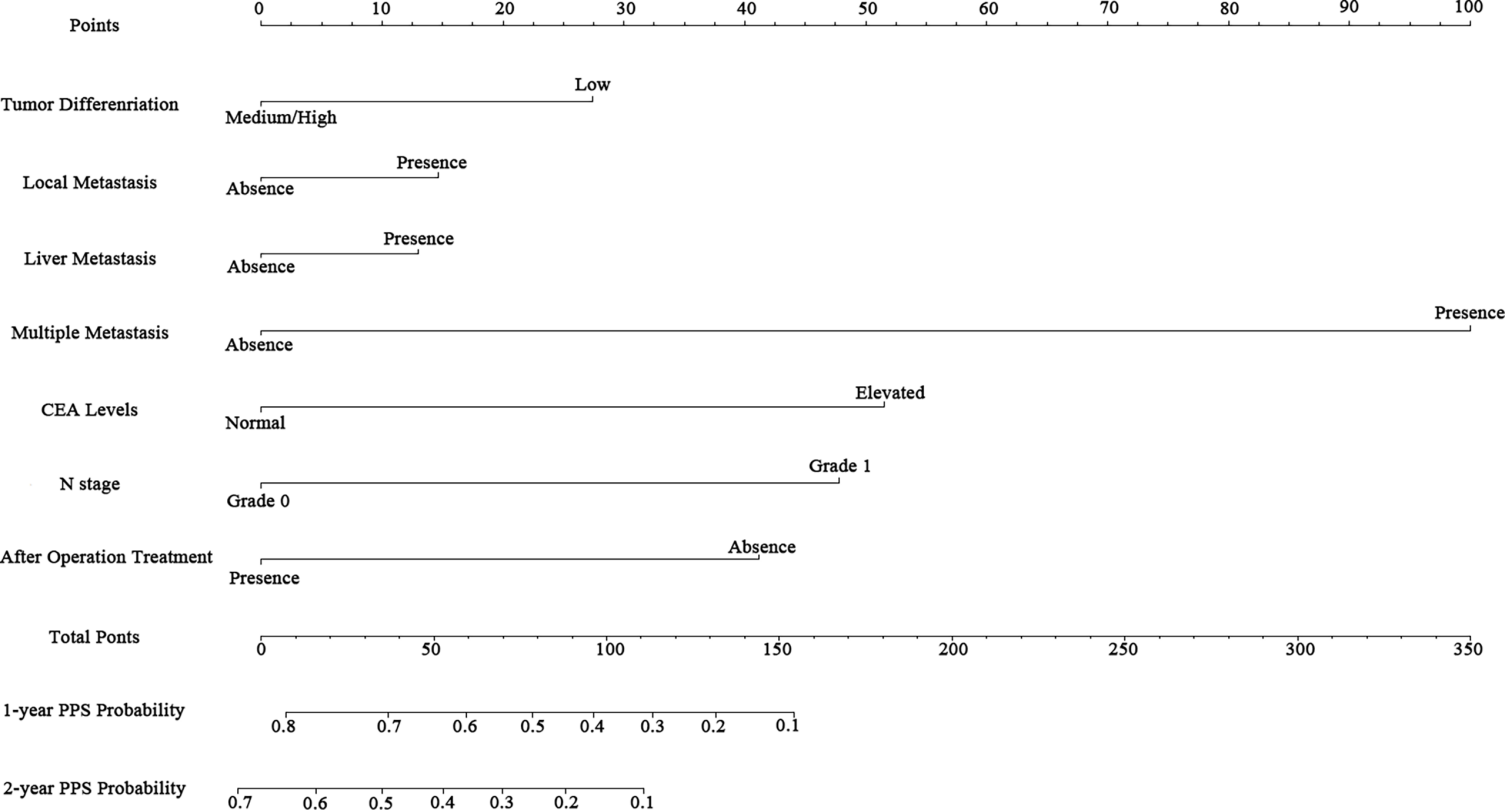
Nomogram for predicting the 1- and 2-year post-progression survival rates in patients with iCCA.

**Figure 4:**
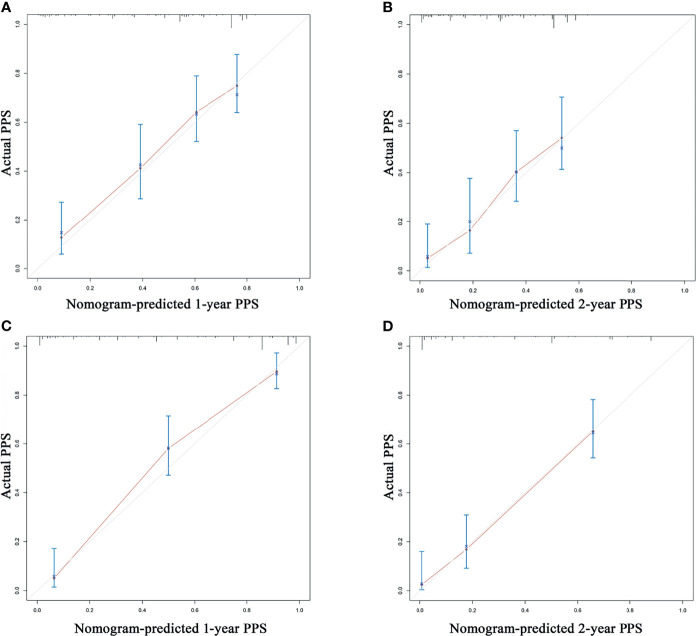
The calibration curve for predicting post-progression survival at 1 year and 2 years in the training cohort **(A, B)** and validation cohort **(C, D)**.

**Figure 5:**
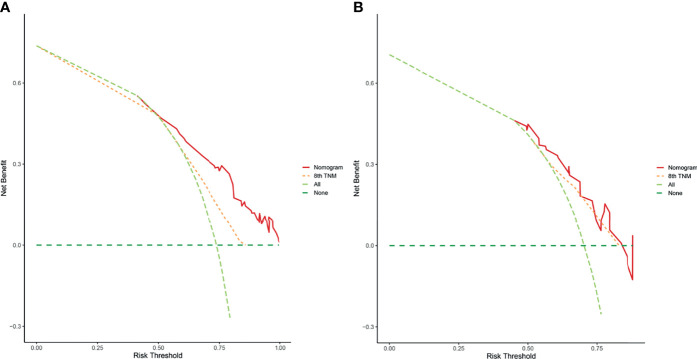
The decision curve analysis of this nomogram for predicting PPS in the primary **(A)** and validation **(B)** cohorts.

**Figure 6:**
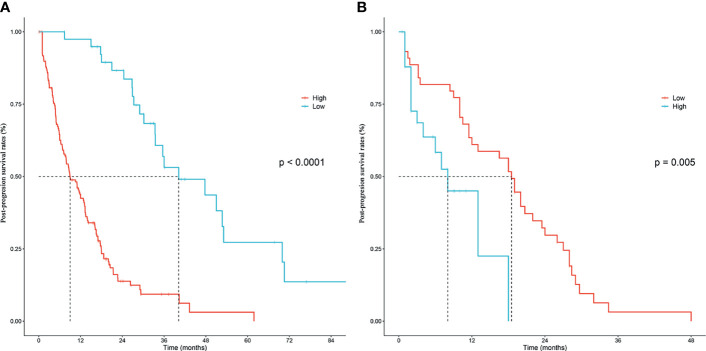
Kaplan–Meier post-progression survival stratified by the nomogram score in the training cohort **(A)** and validation cohort **(B)**, respectively.

According to the independent prognostic factors defined in the multivariable analysis, a nomogram was constructed to predict 1- and 2-year PPS for postoperative progressed iCCA patients (**Figure 3**). This nomogram could evaluate the probability of survival outcomes by adding up the scores for each variable. An objectively high agreement could be observed between actual and predicted survival in the calibration plots of both primary (**Figures 4A, B**) and validation cohorts (**Figures 4C, D**). The C-indexes of the present nomogram in the primary and validation cohorts were 0.794 (95% CI 0.769–0.828) and 0.827 (95% CI 0.532–0.678), respectively; these values were significantly higher than those of the 8th TNM stage system (**Table 3**). To further evaluate the performance of our nomogram, the decision curve analysis was carried out in both the primary and validation cohorts (**Figure 5**); the nomogram also showed more outstanding performance than the 8th TNM staging system did for PPS prediction in recurred iCCA patients. Furthermore, the total point of every patient in the primary cohort was calculated according to the constructed nomogram, then the cutoff point was computed using the R package “cutoff.” All the patients in this study were categorized into High- and Low-risk groups according to the cutoff point of 48. The PPS rates of patients in the low-risk group were significantly higher than those in the high-risk group in both primary (**Figure 6A**) and validation cohorts (**Figure 6B**).

**Table 3:** Comparisons of the C-index with the nomogram and 8th TNM stage system in the primary cohort and validation cohorts.

System		PPS	
		C-index	p
Primary cohort	Nomogram	0.794 (0.769–0.828)	<0.001
	TNM stage	0.577 (0.476–0.674)	
Validation cohort	Nomogram	0.827 (0.779–0.876)	<0.001
	TNM stage	0.605 (0.532–0.678)	

## Discussion

Intrahepatic cholangiocarcinoma is a type of heterogeneous hepatobiliary malignancies with increasing incidence worldwide. Compared to HCC, patients with iCCA often suffer from worse overall survival, progression-free survival, and post-progression survival (12). Even after radical surgical resection, the 5-year survival rate rarely exceeded 30% (13, 14). The primary reason responsible for the poor outcomes is recurrence, which occurred in 50%–70% surgically resected iCCA patients (14–16). There have been several studies that concentrated on evaluation and prediction of postoperative recurrence (15, 17–20). However, there is a lack of research that focused on post-progression survival in iCCA patients. Therefore, it is necessary to establish an efficient tool to evaluate and predict PPS in these patients. In the present study, we analyzed the survival outcomes and the patterns of recurrence in multicenter cohorts of iCCA patients. Independent PPS prognostic predictive factors were then adjusted to the multiple characteristics of the tumor and features of progression. Finally, a novel nomogram that could accurately stratify patients into subgroups with distinct prognosis based on the potential PPS rates was established and validated.

In the last few decades, there have been encouraging progresses in optimizing surgical techniques, developing scheme and medicine of adjuvant therapy, and raising public awareness to the iCCA and its high recurrence rate. Several previous studies have revealed that PPS represented up to two-thirds of patients in breast cancer (21), non-small cell lung cancer (22), and ovarian cancer (23). Clearly, PPS represents an increasing important factor in outcomes of cancer patients. Given the relationship between PPS and progression and adjuvant therapy in this study, PPS could be an independent indicator of the outcomes in recurred iCCA patients.

Progression pattern, defined by the first recurred location, plays a vital role in PPS prediction, especially the metastasis progression patterns which had the highest assignment in our nomogram. This observation indicated that the post-progression survival was primarily determined by recurrence-related factors rather than the primary characteristics of the tumor. In accordance with literatures, in the current study it was observed that liver metastasis accounted for the majority of progression patterns, followed by local LN metastasis and multiple metastases, while local plus liver metastasis contributed to a small proportion of tumor progression (15, 18, 20, 24). The reason why liver metastasis accounted for the majority of patterns may be in connection with the portal vein metastatic slant of iCCA. Liver and local progression patterns shared similar PPS rates and displayed significantly better outcomes when compared to other patterns, while multiple progression patterns resulted in the worst PPS outcome. Notably, there was no difference between liver metastasis and local metastasis in PPS. The median PPS of these two patterns is 12–14 months. It is the belongingness to local isolated progression which liver metastasis and local metastasis have in common. This further indicates that with positive and appropriate adjuvant therapy, local isolated progression, whether single liver metastasis or single local LN metastasis, could lead to a relatively better PPS. On the other hand, local multiple-progression pattern and liver plus local led to a relatively worse PPS survival compared to liver or single local multiple progression pattern, but still much better than multiple-metastasis patterns.

Opposite to the previous studies and our anticipation, progression period, defined as time to recurrence or metastasis, had no significant prognostic prediction value to PPS (11, 18). This result might be interfered by the patients’ compliance as they might not have conducted the check-back schedule regularly. On the other hand, it is worth noting that there are a series of the difficulties in identifying tumor progression by imaging examinations. In addition, receiving adjuvant treatments in the early-progression patients often resulted in better PPS.

Similar to previous studies, we observed in the current studies that CEA, as a vital tumor marker, was an independent prognostic factor for PPS in iCCA patients (25, 26). Elevated CEA levels often indicated higher tumor burden and worse malignant characteristics in iCCA, which has further implication as poor treatment responses. Besides, tumor markers such as CEA may also have powerful prognostic value in predicting the survival of iCCA patients. Further, pathological features of primary tumor are equally crucial as CEA levels in our model. A previous study showed that tumors of poorly histopathologic stage promote the development of metastasis and shorten survival times by secreting molecules such as E-cadherin and epidermal growth factor (27). Whether or not positive lymph nodes were uncovered in pathological diagnosis is also involved in the nomogram. Different from HCC, in which lymph node metastasis were rarely observed, iCCA often spreads through the lymphatic system (19). As a result, intraoperative lymphadenectomy is highly recommended by guidelines and most studies (9, 28).

The potential benefits of postoperative adjuvant therapy in iCCA remained controversial (29). In this present study, we demonstrated for the first time that postoperative adjuvant therapy provided clinical benefits in PPS of iCCA patients. Compared to breast cancer and lung cancer, the treatment responses of iCCA to chemotherapy, targeted therapy, and immunotherapy are shown to be underwhelming in plenty of studies through the past decades (9). However, with the continuous efforts in the development of novel therapeutics, a few recent clinical trials reported that chemotherapy led to improved survival for iCCA patients with lymph node metastasis or advanced tumor stages (30, 31). Similarly, in the current study, significant differences in PPS were observed between patients who did receive postoperative therapies and those who did not. Our results demonstrated that conventional adjuvant therapy benefited the PPS of iCCA patients. As for the patients at high risk for recurrence, the treatment scheme should be adjusted based on clinical observations in more intensive monitoring.

Finally, we constructed a novel nomogram based on these independent prognostic factors and further calculated the cutoff of the total points. In accordance with the cutoff point, patients in primary cohorts and validation cohorts were classified into high-risk and low-risk subgroups, respectively. The patients at high risk demonstrated significantly poorer PPS than those who are at low risk in both cohorts. Therefore, this validated nomogram could accurately stratify patients into subgroups with significantly different PPS rates. Moreover, it may provide assistance with intensive monitoring and determining of adjuvant therapy for recurred iCCA patients.

There were several limitations in the present study. First, although we have studied patients from multicenter cohorts, only four progression patterns were analyzed in this study. An extended follow-up period is warranted to obtain additional information and provide more precise tumor progression patterns after surgical resection. Secondly, the retrospective data can sometimes be obscure since the specific regimens as well as the lengths of the therapy period were unavailable in this study. Thirdly, there are systematic bias of a retrospective study caused by the incomplete adherence to postoperative follow-up protocol. Fourth, the molecular analysis was not studied in the present study; the underlying mechanisms between poor PPS and the significantly prognostic factors were valuable to lucubrate. Last, the sample size of this present study was not numerous enough; further extensive large, trans-regional studies were needed to verify the prognostic power of this novel nomogram.

## Conclusion

To our knowledge, the current study is the first to construct a nomogram for PPS of recurred iCCA patients in multicenter patient cohorts. We analyzed the PPS of different progression patterns of iCCA and further established a novel nomogram to predict PPS in postoperative recurred iCCA patients. In addition to the primary tumor features, the inclusion of progression patterns ensured the better prognostic prediction by this nomogram. Besides, the current study was conducted in diverse patient populations from the multicenter cohorts, which further strengthened the predictive power of the novel nomogram. Therefore, this novel nomogram could help clinicians to predict the PPS of recurred iCCA patients. The patients which were classified into the high-risk group by this nomogram should be monitored more frequently. Early and regular adjuvant therapy would also benefit those patients with high risk in this nomogram.

## Data Availability Statement

The raw data supporting the conclusions of this article will be made available by the authors, without undue reservation.

## Ethics Statement

Ethical approval/written informed consent was not required for the study of animals/human participants in accordance with the local legislation and institutional requirements.

## Author Contributions

Study concept: XL. Study design: CZ, CH, JL. Drafting of the manuscript: CZ, CH, JL. Data collection: CZ, CH, JL, CC, XH. Data analysis: CH, CZ, JL, XH. Critical revision of the manuscript: XL. All authors contributed to the article and approved the submitted version.

## Funding

This work was supported by grants from the National Natural Science Funds (No. 82102166), Guangdong Basic and Applied Basic Research Foundation (2020A1515110954), and Sun Yat-sen University Grant for Medical Humanities Practice and Teaching (No. 23000-18008023).

## Conflict of Interest

The authors declare that the research was conducted in the absence of any commercial or financial relationships that could be construed as a potential conflict of interest.

## Publisher’s Note

All claims expressed in this article are solely those of the authors and do not necessarily represent those of their affiliated organizations, or those of the publisher, the editors and the reviewers. Any product that may be evaluated in this article, or claim that may be made by its manufacturer, is not guaranteed or endorsed by the publisher.
