# Supplementary material for: Progression Patterns and Post-Progression Survival in Recurred Intrahepatic Cholangiocarcinoma Patients: A Novel Prognostic Nomogram Based on Multicenter Cohorts
Source: Front Oncol. 2022 Apr 8;12:832038. doi: 10.3389/fonc.2022.832038 (PMC9033166; doi:10.3389/fonc.2022.832038)
Supplement: Supplementary file 1 [file Table_1.docx]

| Table S1. Clinical and pathological characteristics of all iCCA patients in the primary cohort (SYSUCC cohort) and validation cohort (FHDMU cohort). | | | | | |
| --- | --- | --- | --- | --- | --- |
| **Variables** | **Primary cohort**  **(n=289)** | **Validation cohort**  **(n=107)** | **Variables** | **Primary cohort**  **(n=289)** | **Validation cohort**  **(n=107)** |
| Gender |  |  | Macrovascular invasion |  |  |
| Male | 178 (61.6%) | 62 (57.9%) | Absence | 271 (93.8%) | 95 (88.8%) |
| Female | 111 (38.4%) | 45 (42.1%) | Presence | 18 (6.23%) | 12 (11.2%) |
| Age (years) |  |  | Satellite sites |  |  |
| ≤60 years | 189 (65.4%) | 33 (30.8%) | Absence | 198 (68.5%) | 106 (99.1%) |
| >60 years | 100 (34.6%) | 74 (69.2%) | Presence | 91 (31.5%) | 1 (0.93%) |
| WBC count (×10^9^/L) |  |  | Adjacent Organ Invasion |  |  |
| ≤10 | 256 (88.6%) | 92 (86.0%) | Absence | 257 (88.9%) | 103 (96.3%) |
| >10 | 33 (11.4%) | 15 (14.0%) | Presence | 32 (11.1%) | 4 (3.74%) |
| HGB (g/L) |  |  | Tumor size |  |  |
| ≤175 | 125 (43.3%) | 30 (28.0%) | ≤ 5cm | 112 (38.8%) | 52 (48.6%) |
| >175 | 164 (56.7%) | 77 (72.0%) | ≤ 5cm | 177 (61.2%) | 55 (51.4%) |
| PLT (×109/L) |  |  | LN metastasis |  |  |
| ≤350 | 10 (3.46%) | 5 (4.67%) | Absence | 247 (85.5%) | 95 (88.8%) |
| >350 | 279 (96.5%) | 102 (95.3%) | Presence | 42 (14.5%) | 12 (11.2%) |
| ALT (U/L) |  |  | Positive LN number: |  |  |
| ≤50 | 251 (86.9%) | 55 (51.4%) | 0 | 247 (85.5%) | 95 (88.8%) |
| >50 | 38 (13.1%) | 52 (48.6%) | 1 | 18 (6.23%) | 3 (2.80%) |
| AST (U/L) |  |  | 2 | 10 (3.46%) | 4 (3.74%) |
| ≤40 | 251 (86.9%) | 56 (52.3%) | 4 | 6 (2.08%) | 2 (1.87%) |
| >40 | 38 (13.1%) | 51 (47.7%) | 5 | 4 (1.38%) | 2 (1.87%) |
| ALP (U/L) |  |  | 6 | 3 (1.04%) | - |
| ≤ 125 | 179 (61.9%) | 25 (23.4%) | 9 | - | 1 (0.93%) |
| > 125 | 110 (38.1%) | 82 (76.6%) | 12 | 1 (0.35%) | - |
| GGT (U/L) |  |  | Tumor differentiation |  |  |
| ≤ 60 | 106 (36.7%) | 16 (15.0%) | Low | 32 (11.1%) | 3 (2.80%) |
| > 60 | 183 (63.3%) | 91 (85.0%) | Medium/High | 257 (88.9%) | 81 (75.7%) |
| ALB (g/L) |  |  | T stage 8th |  |  |
| > 40 | 4 (1.4%) | 38 (35.5%) | 1 | 68 (23.5%) | 84 (78.5%) |
| ≤ 40 | 285 (98.7%) | 69 (64.5%) | 2 | 44 (15.2%) | 5 (4.67%) |
| TBIL (μmol/L) |  |  | 3 | 153 (52.9%) | 14 (13.1%) |
| ≤ 20.5 | 262 (90.7%) | 54 (50.5%) | 4 | 24 (8.30%) | 4 (3.74%) |
| > 20.5 | 27 (9.34%) | 53 (49.5%) | N stage 8^th^ |  |  |
| IBIL (μmol/L) |  |  | Absence | 247 (85.5%) | 89(83.1%) |
| ≤ 15 | 272 (94.1%) | 65 (60.7%) | Presence | 42 (14.5%) | 18(16.9%) |
| > 15 | 17 (5.88%) | 42 (39.3%) | TNM 8^th^ |  |  |
| HBsAg |  |  | IA | 31 (10.7%) | 35 (32.7%) |
| Absence | 160 (55.4%) | - | IB | 36 (12.5%) | 46 (43.0%) |
| Presence | 129 (44.6%) | - | II | 37 (12.9%) | 2 (1.87%) |
| CA19-9 (U/ml) |  |  | IIIA | 125 (43.3%) | 8 (7.48%) |
| ≤ 35 | 140 (48.4%) | 25 (23.4%) | IIIB | 60 (20.8%) | 16 (15.0%) |
| >35 | 149 (51.6%) | 82 (76.6%) | After operation therapy |  |  |
| CEA (ng/ml) |  |  | Absence | 159 (55.0%) | 72 (67.3%) |
| ≤ 5 | 208 (72.0%) | 60 (56.1%) | Presence | 130 (45.0%) | 35 (32.7%) |
| > 5 | 81 (28.0%) | 47 (43.9%) | LN5 metastasis |  |  |
| NLR |  |  | Absence | 288 (99.7%) |  |
| < 2.62 | 191 (66.1%) | 36 (33.6%) | Presence | 1 (0.35%) |  |
| ≥ 2.62 | 98 (33.9%) | 71 (66.4%) | LN7 metastasis |  |  |
| PLR |  |  | Absence | 284 (98.3%) | 106 (99.1%) |
| < 104.85 | 169 (58.5%) | 24 (22.4%) | Presence | 5 (1.73%) | 1 (0.93%) |
| ≥ 104.85 | 120 (41.5%) | 83 (77.6%) | LN8 metastasis |  |  |
| SII |  |  | Absence | 280 (96.9%) | 101 (94.4%) |
| 0 | 66 (22.8%) | 30 (28.0%) | Presence | 9 (3.10%) | 6 (5.60%) |
| 1 | 223 (77.2%) | 77 (72.0%) | LN9 metastasis |  |  |
| LCR |  |  | Absence | 283 (97.9%) | - |
| 0 | 21 (7.27%) | - | Presence | 6 (2.08%) | - |
| 1 | 268 (92.7%) | - | LN12 metastasis |  |  |
| PNI |  |  | Absence | 262 (90.7%) | 96 (89.7%) |
| 0 | 274 (94.8%) | 48 (44.9%) | 1 | 23 (7.96%) | 8 (7.48%) |
| 1 | 15 (5.19%) | 59 (55.1%) | 2 | 3 (1.04%) | 2 (1.87%) |
| PI |  |  | 4 | 1 (0.35%) | - |
| 0 | 217 (75.1%) | 32 (29.9%) | 5 | - | 1 (0.93%) |
| 1 | 61 (21.1%) | 63 (58.9%) | LN13 metastasis |  |  |
| 2 | 11 (3.81%) | 12 (11.2%) | Absence | 281 (97.3%) | 102 (95.3%) |
| mGPS |  |  | Presence | 8 (2.7%) | 5 (4.7%) |
| 0 | 230 (79.6%) | 35 (32.7%) | LN14 metastasis |  |  |
| 1 | 56 (19.4%) | 42 (39.3%) | Absence | 288 (99.7%) | - |
| 2 | 3 (1.04%) | 30 (28.0%) | Presence | 1 (0.35%) | - |
| Microvascular invasion |  |  | LN16 metastasis |  |  |
| Absence | 234 (81.0%) | 86 (89.7%) | Absence | 286 (99.0%) | - |
| Presence | 55 (19.0%) | 11 (10.3%) | Presence | 3 (1.04%) | - |
| Lymph-vessel invasion |  |  |  |  |  |
| Absence | 270 (93.4%) | - |  |  |  |
| Presence | 19 (6.57%) | - |  |  |  |
